# Sarcopenia is associated with lower quality of life scores among community-dwelling older Filipinos: Findings from a cross-sectional study

**DOI:** 10.1016/j.tjfa.2025.100044

**Published:** 2025-04-30

**Authors:** Robby Carlo Tan, Kyler Kenn Castilla, Michael Serafico, Marco Mensink, Lisette de Groot

**Affiliations:** a Department of Science and Technology, Food and Nutrition Research Institute, Taguig City, Metro Manila, Philippines; b Division of Human Nutrition & Health, Wageningen University and Research, Wageningen, The Netherlands

**Keywords:** Aging, Older adults, Philippines, Quality of life, Sarcopenia

## Abstract

**Background and Objectives:**

Sarcopenia, characterized by a progressive decline in muscle mass and strength, is a significant concern among older individuals, impacting their functionality and overall quality of life (QOL). The relationship between sarcopenia and QOL among Filipino older adults remains underexplored. Thus, the study aims to determine the prevalence of sarcopenia and its association with the QOL of community-dwelling older adults in selected Philippine cities.

**Design:**

This cross-sectional study used convenience sampling in the selection of the cities. Participants were randomly selected from the list provided by each local city government.

**Setting and Participants:**

536 apparently healthy community-dwelling Filipino older adults from three major islands in the Philippines.

**Methods:**

Sarcopenia was determined using the 2019 Asian Working Group for Sarcopenia criteria which is the presence of low muscle mass, plus low muscle strength, and/or low physical performance. Quality of life was assessed using the culturally-validated WHO-QOL short form questionnaire. Mixed Model logistic regression adjusted for covariates was performed to study the association of sarcopenia indicators to quality of life.

**Results:**

24.3 % of older Filipinos were found to have sarcopenia. Sarcopenic community-dwelling older adults had significantly decreased odds of attaining higher score in the physical domain (OR 0.63; 0.40–0.98), psychological domain (OR 0.55; CI 0.35–0.84), and overall quality of life (OR 0.57; CI 0.37–0.89) than the non-sarcopenic group.

**Conclusion:**

One in four community-dwelling Filipino older adults met the sarcopenia criteria. Sarcopenia is associated with decreased QOL scores, particularly in the physical health and psychological health domains. Sarcopenia needs to be considered in the context of public health to come up with targeted nutrition and health interventions for improved QOL.

**List of abbreviations**
ASM: Appendicular Skeletal Muscle Mass
ASMI: Appendicular Skeletal Muscle Mass Index
AWGS: Asian Working Group for Sarcopenia
BIA: bioelectrical Impedance Analysis
BMI: Body Mass Index
CGA: Comprehensive Geriatric Assessment
DXA: Dual-energy X-ray absorptiometry
HGS: Handgrip strength
HRQOL: Health-related quality of life
PP: Physical performance
QOL: Quality of Life
SARQoL: Sarcopenia Quality of Life
SPPB: Short Physical Performance Battery
SPSS: Statistical Package for Social Sciences
WHO: World Health Organization
WHOQOL-BREF: World Health Organization Quality of Life Brief Version

## 1 Introduction

Older adults comprised 8.6 % of the Filipino population in 2020 and this proportion is expected to increase to 16.5 % by 2050 [1]. As the demography shifts to an aging society, the older population are met with an increased susceptibility to chronic conditions, morbidity, and mortality. It is well-established that a decline in muscle mass accompanies the physiological changes caused by aging [2,3]. This decline has been linked to reduced physical function, leading to increased dependence in performing activities of daily living [[4], [5]–6].

Sarcopenia is defined as the gradual loss of skeletal muscle mass with age accompanied by loss of muscle strength and/or reduced physical performance [7]. A systematic review and meta-analysis of 151 studies reported 10 % to 27 % global prevalence for sarcopenia among those aged 60 years and above [8]. Variations in prevalence relied considerably on classifications and cut-off points used. In 2019, the Asian Working Group for Sarcopenia (AWGS 2019) updated the consensus on the diagnosis in community and clinical settings for sarcopenia among Asian populations. Using the AWGS 2019 algorithm, a study conducted among Thai community-dwelling older adults aged 60 years and above found a 22.2 % and 9.4 % prevalence for sarcopenia and severe sarcopenia, respectively [9]. Also, a cohort among Malaysian older adults reported a prevalence of 5.0 % for sarcopenia, and 3.6 % for severe sarcopenia [10]. Despite the growing recognition of sarcopenia in neighboring Southeast Asian countries, large-scale epidemiological studies on its prevalence remain scarce in the Philippines.

In addition to prevalence, predictors and consequences of sarcopenia is imperative for prevention, early diagnosis and proper intervention. Studies conducted in Southeast Asian countries found associations between age, body mass index (BMI), and waist circumference towards sarcopenia [11,12]. Research among Koreans further highlighted that older adults with sarcopenia had significantly lower health-related quality of life (HRQOL) compared to their non-sarcopenic counterparts [13]. Similarly, a meta-analysis by Beaudart et al. revealed that sarcopenic individuals had significantly lower HRQOL as compared to those without sarcopenia [14]. In addition to reduced QOL, sarcopenia is associated with several adverse outcomes such as higher rate of mortality, falls, and hospitalization [15]. All these, underscores the far-reaching implications of sarcopenia, reinforcing the need to investigate its impact to the overall QOL of an older individual.

QOL is an important indicator of health and well-being in later life. Evidence from previous studies suggests that older adults with sarcopenia may experience limitations that extend beyond physical impairment, potentially affecting their mental and social aspects in life. It is hypothesized that older Filipino adults with sarcopenia will show significantly lower overall QOL compared to their non-sarcopenic counterparts. However, despite its known impact on daily function and independence, sarcopenia remains underexplored among Filipino older adults.

Hence, this study aims to determine the prevalence of sarcopenia and examine its association with the physical, psychological, social, and environmental domains of QOL among community-dwelling older adults in selected cities in the Philippines. As the country anticipates a demographic shift toward an aging population, this study can contribute to an early formulation of age-sensitive policies and targeted interventions, ultimately contributing to the overall well-being and QOL of the Filipino older population. Additionally, findings from this research may offer valuable insights for other countries with similar demographic trends, aiding in the pro-active formulation of strategies to address sarcopenia and promote healthy aging on a broader scale.

## 2 Methods

### 2.1 Study Design, study site and study participants

This paper is part of the Healthy Aging Program for Senior Citizens (HAPPY Senior Citizens study), a cross-sectional study among community-dwelling Filipino older adults that was conducted from November 2021 to June 2022 at three different cities in the Philippines namely Tarlac, Tacloban and Davao. At the time of the data collection, the study sites were under COVID-19 pandemic under Alert Level 2 status which limits the face-to-face gathering to a maximum of 50 % and 70 % for indoor and outdoor venues, respectively [16] and strict intercity travel in the country. One city from each major island province in the country namely Luzon, Visayas, and Mindanao was chosen as study site based on the presence and proximity of geriatricians or physicians trained in the conduct of Comprehensive Geriatric Assessment (CGA) by the Institute on Aging - National Institutes of Health.

**Fig. 1:**
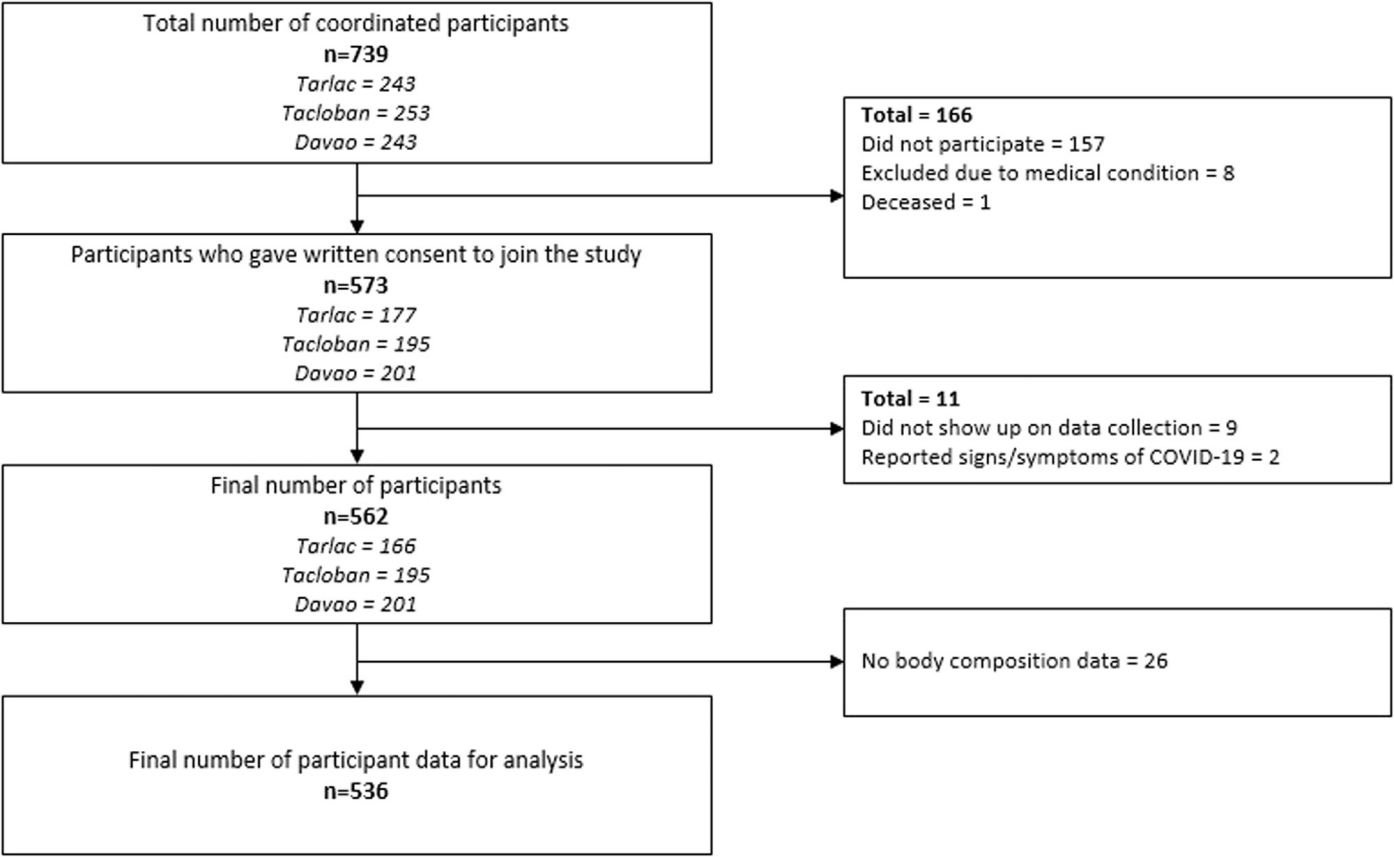
Participant Recruitment and Screening Flowchart.

Local government units with jurisdiction over the cities of Tarlac, Tacloban and Davao were coordinated and requested to identify potential *barangays*, the smallest unit of administrative division, for participant recruitment. Considerations for data collection were primarily based on the total population, safety, feasibility, and accessibility. Four *barangays* were randomly selected in each city to be part of the study sites. Through the local organization for older adults, a list of residents aged 60 and over from each *barangay* was acquired. Initially, 200 older adults were randomly sampled for each city. The randomly sampled list of older adults was coordinated and verified to each *barangay* through the aid of local health workers. The refusal or absence of the randomly drawn participant was replaced by another participant from the same *barangay* provided that the replacement is within the similar age range and sex. Apparently healthy community-dwelling older adults aged 60 years and older that can participate independently in the research activities and provide informed consent were included. Meanwhile, being bedridden, unable to comprehend and answer interview questions, and presence of severe illness/condition (e.g. cancer, transplant) were the exclusion criteria. All participants provided informed consent after thorough explanation of the research study. From the 562 participants, 26 did not have body composition data. Fig. 1 shows the coordination, recruitment and screening process of the participants. A total of 536 were included in the analysis.

### 2.2 Sample Size calculation

G*Power software version 3.1 was used to calculate the sample size by city using a priori analysis to determine the minimum sample size based on the 22.0 % prevalence of sarcopenia among Malaysian community-dwellers aged 60 years and above [5], 95 % confidence level, and a 5 % margin of error. A minimum sample size of 264 for the entire study to derive relevant implications on the research topic. Accounting for 30 % drop out rate and absence of complete randomization at the site selection, a maximum of 600 participants or 200 per city was targeted for the entire study.

### 2.3 Measurement Variables

#### 2.3.1 Anthropometry and body composition

Weight and body composition were measured using a multi-frequency (8 electrodes) segmental body impedance analyzer (BIA) following the manufacturer’s protocol (Tanita MC-780MA, Kowloon, HK). The BIA equipment was set on flat, solid, and sturdy ground. Participants were asked to wear minimal clothing without any metal accessory, while in a fasted state or with minimal food and liquid intake. Participants were asked to stand barefoot on the feet electrodes in the platform; arms not touching the sides of the body and hands gripping the left and right handles. Information on fat mass, fat-free mass (total and segmental), body water, impedance, and phase angle were generated and saved using the Tanita GMON Software. Appendicular Skeletal Muscle Mass (ASM) was the total muscle mass of both arms and legs. Appendicular Skeletal Muscle Mass Index (ASMI) was calculated by dividing ASM by the height in meters squared. BMI was used as the indicator of nutritional status using the WHO classification [17]. Height was measured using a portable stadiometer (SECA 213, Chino, USA) without any footwear and headwear. Calf circumference was measured using a retractable, non-stretchable tape (SECA 201, Chino, USA).

Two measurements were taken with each reading agreeing to be within 0.5 cm. A third measurement was taken when the first two readings do not agree. Then, the average of the measurement was taken as the final measurement.

#### 2.3.2 Handgrip strength

Handgrip Strength (HGS) was determined using a hand-held dynamometer (Jamar® Hydraulic Hand Dynamometer, USA). Participants were asked to remove any wrist or hand accessories as they sat comfortably in a chair with back support, and without armrests. Shoulders were adducted and neutrally rotated, elbow flexed at 90 degrees, forearm in neutral position, and the wrist positioned in slight extension. The dominant and non-dominant hand of the participant was confirmed through the questionnaire prior to the HGS test. Three alternating handgrip measurements, with 30 s of rest after each press, were recorded for both hands. The highest value of the dominant hand was used for the analysis [18].

#### 2.3.3 Physical performance

Balance, gait speed, and lower leg strength through chair stand test were collected for the Short Physical Performance Battery (SPPB) Test to assess physical performance [19,20]. For the balance subcomponent, the participant maintained three different stances (side-by-side stance, semi-tandem stance, and tandem stance) for a maximum of 10 s each. Participants were assessed on the duration they could maintain each stance. To measure gait speed, participants were timed to walk along a 4-meter pathway at a normal speed, twice. The shorter time among the two measurements was noted as the final time. Participants were timed to complete sit-to-stand movements repeated five times for the chair stand Test. The SPPB is evaluated under a score system and each subcomponent has a different criterion to acquire a certain score [21]. Each subcomponent has a maximum of 4 points each, making a total of 12 points for the entire SPPB test.

#### 2.3.4 Presence of sarcopenia

The presence of sarcopenia was determined using the AWGS 2019 criteria, which include three key components: (1) low muscle mass, indicated by an ASMI of < 7.0 kg/m² for men and < 5.7 kg/m² for women, measured via bioelectrical impedance analysis (BIA); (2) low muscular strength, defined as a handgrip strength of < 28 kg for men and < 18 kg for women; and (3) low Physical Performance (PP), determined by *An sppb* score of ≤ 9 or a chair stand test time of ≥ 12 s. Severe sarcopenia was diagnosed when all three criteria were present, whereas the presence of low muscle mass combined with either low muscle strength or low physical performance indicated sarcopenia. In this study, participants were first screened in community preventive service settings to identify those at risk of sarcopenia before proceeding to formal diagnosis [7].

#### 2.3.5 Quality of life

The perception of older adults on their overall quality of life (QOL) was assessed using the World Health Organization Quality of Life Brief Version (WHOQOL-BREF) questionnaire [22]. The questionnaire used in this study was culturally-validated among older Filipinos [23]. The 26-item questionnaire covers four domains of life including physical health, psychological health, social relationships, and environment. Each item was scored through a 5-point ordinal scale dependent on the participant’s degree of perceived capability, frequency or contentment on specific facets of their life within the last two weeks. The 20-point scoring process was used for each domain and the sum of the four domains is the overall total score. Higher score indicated higher QOL perception. The computation was based on the WHOQOL-BREF manual [24].

#### 2.3.6 Demographics, lifestyle and health information

Age, sex, civil status, household size, and educational attainment were gathered through an interview method. Lifestyle and health-related information, specifically smoking status (current or not), alcohol consumption (current or not), medications used in the past two weeks (yes or no), and health care arrangements were recorded. Perceived ability to financially support healthcare needs was used as an indicator of financial readiness. Physical activity level was assessed using the Global Physical Activity Questionnaire. In a rested state, blood pressure was measured using a non-mercurial sphygmomanometer (A&D UM-102A, Oxfordshire, UK).

### 2.4 Statistical analyses

Continuous data were checked for normality distribution through visual plot and Shapiro-Wilk Test. Descriptive data were reported as mean and standard deviation or median and interquartile range while categorical data were expressed in count and percentages. The difference between sarcopenic and non-sarcopenic groups was determined using Student *t*-test for equal variances while Welch's *t*-test for unequal variances. Chi-square test was used for categorical variables.

The dependent variables are the score of the QOL per domain and overall. This was classified as high (66th percentile and above) and low (below the 66th percentile). Sarcopenia and its criteria were the independent variable. Analysis on the association of QOL scores between sarcopenia and non-sarcopenia groups was determined using mixed model binary regression with city and *barangays* as random effects. Analysis was adjusted for age, sex, smoking, alcohol consumption, financial readiness, and medications taken.

Additionally, participants were grouped into three based on the absence or presence and combination of sarcopenia criteria. The groupings were absence of all sarcopenia criteria or presence of only one criterion (low HGS *or* low physical performance or low ASMI) as the normal reference group (Group A); those with both low HGS and low PP but not classified as sarcopenic based on the AWGS 2019 criteria (Group B); and those with sarcopenia and severe sarcopenia (sarcopenia group). Characteristics of the three groups were analyzed using ANOVA for continuous value and chi-square test for categories. Mixed model multinomial regression was used to identify the association of QOL scores across the three groups and was adjusted using the same covariates as the mixed model binary regression. All analyses were done using Statistical Package for Social Sciences (version 28, IBM, Armonk, NY, USA). Results with *p* value of < 0.05 were considered as statistically significant.

## 3 Results

### 3.1 Sarcopenia prevalence

**Fig. 2:**
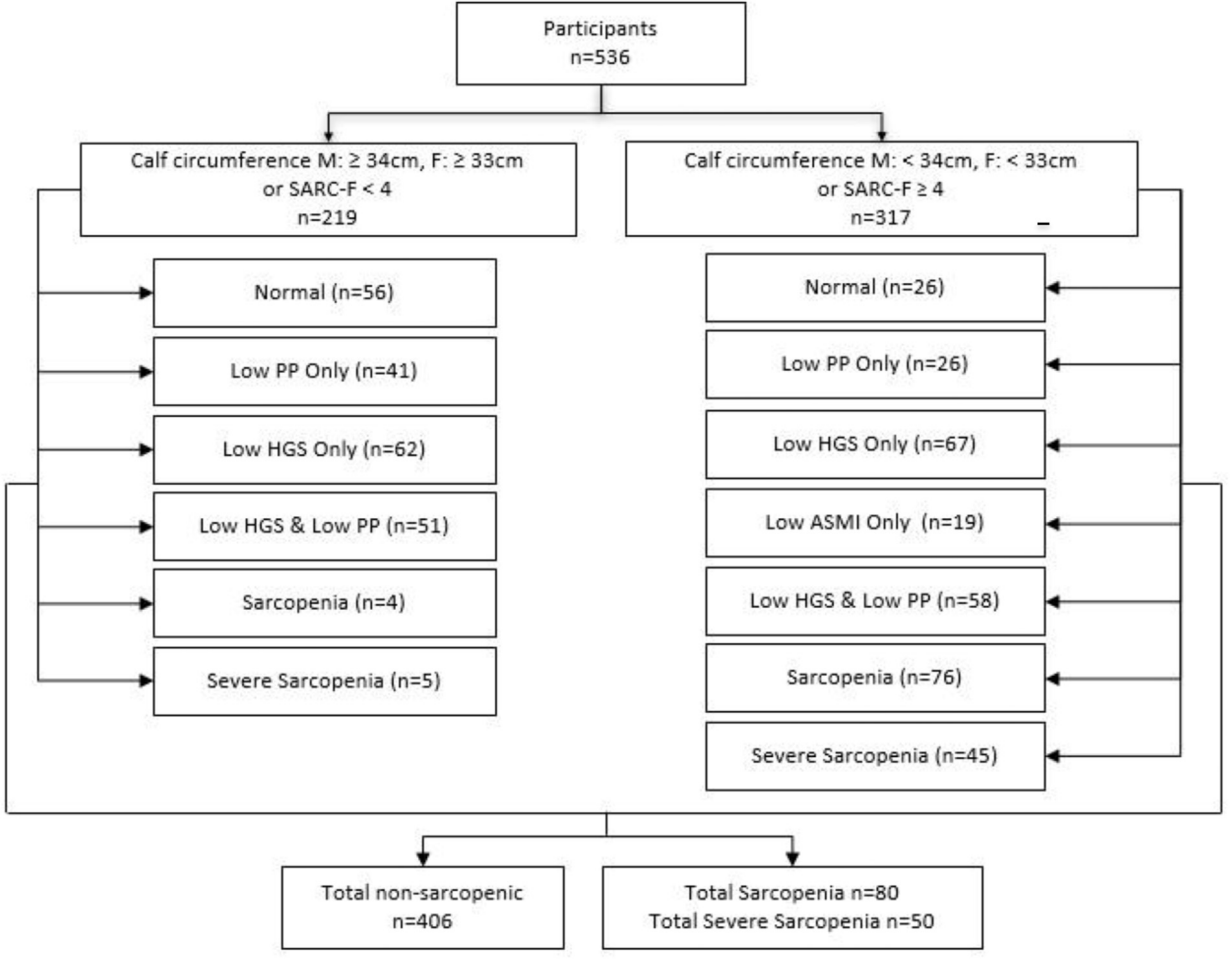
Diagnosis of Sarcopenia among community-dwelling older Filipinos according to the AWGS 2019 algorithm.

As shown in Fig. 2, After screening for those at-risk for sarcopenia in the community setting, a total of 317 (59.1 %) were found to have either low calf circumference or a SARC-F score of ≥ 4. Diagnosis following the criteria set for ASMI, HGS, and physical performance resulted in 76 sarcopenic and 45 severely sarcopenic participants in this group. In general, the high number of older adults with low HGS was remarkable in both the risk and non-risk groups. Among the 219 non-risk participants (calf circumference M: ≥ 34 cm, F: ≥ 33 cm or SARC-*F* < 4), it was found that four participants were sarcopenic while five were severely sarcopenic. Notably, 387 out of the total participant (72.2 %) manifest presence of Low HGS, solely or in combination with other sarcopenia criteria. The overall total of participants with sarcopenia based on the AWGS 2019 criteria was 24.3 % (*n* = 130) of which 9.3 % (*n* = 50) participants had severe sarcopenia.

### 3.2 Overall characteristics

Characteristics of sarcopenic and non-sarcopenic group are shown in Table 1. The overall median age was 68.1 ± 8.3 years. A significant difference in median age was observed between groups with the sarcopenia group being older (72.3 ± 11.3 yr) than the non-sarcopenia group, (66.8 ± 7.6 yr). Overall, there were more female participants (61.2 %) than male (38.8 %). In terms of sarcopenia, prevalence was at 31.2 % for the entire male participants while 19.8 % for females. No significant differences were found in the number of household members living with, smoking status, alcohol consumption, and number of medications taken in both groups. The sarcopenia group had significantly lower BMI, calf circumference, ASMI, HGS for both hands, SPPB score than the non-sarcopenia group.

**Table 1:** Characteristics of sarcopenic and non-sarcopenic participants according to the AWGS 2019.

Variables	Overall (*n* = 536)	Non-Sarcopenia (*n* = 406)	Sarcopenia (*n* = 130)	p-value
Age (y)	68.1 (8.3)	66.8 (7.6)	72.3 (11.3)	**<0.001**
Sex (%) Male Female	208 (38.8) 328 (61.2)	143 (68.7) 263 (80.2)	65 (31.2) 65 (19.8)	**<0.003**
Educational Level (%)^#^ Elementary Highschool College	221 (41.5) 185 (34.8) 126 (23.7)	153 (69.2) 148 (80.0) 101 (80.2)	68 (30.8) 37 (20.0) 25 (19.8)	**<0.016**
No. of hh members (%)^#^ 1 to 3 4 to 6 7 or more	201 (37.6) 194 (36.3) 139 (26.0)	144 (71.6) 148 (76.3) 112 (80.6)	57 (28.4) 46 (23.7) 27 (19.4)	0.163
Current smoker (%)	73 (13.6)	54 (74.0)	19 (26.0)	0.270
Current alcohol consumer (%)	194 (36.2)	153 (78.9)	41 (21.1)	0.059
Met the PA recommendation (%)^#^	388 (78.2)	306 (78.9)	82 (21.1)	**<0.040**
No. of medication taken (%) 0 1–2 3 or more	216 (40.3) 233 (43.5) 87 (16.2)	156 (72.2) 183 (78.5) 67 (77.0)	60 (27.8) 50 (21.5) 20 (23.0)	0.283
Body Mass Index (kg/m^2^)	24.1 ± 4.4	25.2 ± 3.9	20.7 ± 3.9	**<0.001**
Calf Circumference (cm)	32.7 ± 3.5	33.6 ± 3.1	29.8 ± 2.9	**<0.001**
ASMI (kg/m^2^)	6.5 (1.2)	6.8 (1.2)	5.6 (1.0)	**<0.001**
Total body fat (%)	31.9 (14.7)	33.3 (13.8)	27.1 (15.4)	**<0.001**
Handgrip Strength, dominant (kg)	18.0 (10.8)	18.0 (11.0)	14.3 (8.3)	**<0.001**
Handgrip Strength, non-dominant (kg) ^#^	16.0 (10.0)	17.0 (12.0)	14.0 (9.0)	**<0.001**
SPPB Score^#^	11.0 (2.0)	11.0 (2.0)	11.0 (3.0)	**<0.001**
Chairstand Score (seconds) ^#^	11.6 (4.3)	11.6 (4.2)	11.8 (4.9)	0.346
Quality of Life Score				
Physical Health Domain	16.0 (2.9)	16.6 (2.9)	16.0 (2.4)	**0.030**
Psychological Health Domain	16.7 (2.7)	16.7 (2.7)	16.0 (2.7)	**<0.001**
Social Relationship Domain^#^	18.0 (4.0)	18.0 (4.0)	17.3 (2.7)	0.159
Environmental Domain	17.0 (3.0)	17.0 (2.5)	17.0 (3.0)	0.096
Overall Score^#^	67.5 (9.1)	68.1 (8.7)	65.7 (10.8)	**<0.003**

Values presented are mean ±SD, median(IQR), count( %).

ASMI - Appendicular Skeletal Muscle Mass Index; SPPB - Short Physical Performance Battery; QOL- Quality of Life.

#Missing values: Non-sarcopenia (Educational level = 4, No. of hh members = 2, Physical activity = 25, Non-dominant handgrip strength = 1, SPPB score = 10, Chairstand = 3, Social Relationship Domain = 1, Overall QOL = 1); Sarcopenia (Physical activity = 15, SPPB score = 7, Chairstand = 4).

For the additional analysis, the characteristics of group A (reference group), group B (low HGS and low PP), and sarcopenia group are shown in Appendix A. Generally, the reference group showed higher mean in Calf Circumference, ASMI, HGS, PP, and QOL scores as compared to group B and the sarcopenia group.

### 3.3 Sarcopenia and QOL

In terms of the QOL, the physical domain, psychological domain, and the overall score were significantly different between the groups, with the sarcopenia group exhibited significantly lower scores than the non-sarcopenia group (). No significant differences were found in the social relationship and environmental domain scores between the groups.

The results of the mixed model binary regression analysis (Table 2) revealed the sarcopenia group has decreased odds to attain a high overall QOL score (OR 0.57; CI 0.37–0.89), as well as in the physical health (OR 0.63; CI 0.40–0.98) and psychological health domains (OR 0.55; CI 0.35–0.84). For the mixed model multinomial regression analysis as reflected in Table 3, it was observed that group B (physical impaired, non-sarcopenic) and the sarcopenic group has a consistent trend for a decreased odds of attaining higher scores in the physical (OR 0.59; CI 0.37–0.95 / OR 0.54; CI 0.34–0.86), psychological (OR 0.52; CI 0.33–0.83 / OR 0.45; CI 0.28–0.71), and overall QOL scores (OR 0.47; CI 0.29–0.73 / OR 0.46; CI 0.30–0.75) as compared to the group A (reference group). No consistent significant association was observed in the social relationship and environmental domains.

**Table 2:** Odds Ratio of QOL scores per domain and overall by sarcopenia and non-sarcopenia group using mixed model binary regression.

	Quality of Life domains				
	Physical health	Psychological health	Social relationship	Environmental	Overall QOL score
Non-sarcopenia (ref) vs Sarcopenia Group	**0.63 (0.40–0.98)**	**0.55 (0.35–0.84)**	0.89 (0.58–1.38)	0.89 (0.58–1.38)	**0.57 (0.37–0.89)**

Adjusted for age, financial readiness, smoking status, alcohol drinkers, and number of medications taken. Values in **bold** are significant.

**Table 3:** Odds Ratio of QOL scores per domain and overall by sarcopenia and its criteria using mixed model multinomial regression.

	Quality of Life Domains				
	Physical health	Psychological health	Social relationship	Environmental	Overall QOL score
Group A (reference group)					
Group B	**0.59 (0.37–0.95)**	**0.52 (0.33–0.83)**	**0.57 (0.37–0.91)**	0.75 (0.47–1.20)	**0.47 (0.29–0.73)**
Sarcopenia Group	**0.54 (0.34–0.86)**	**0.45 (0.28–0.71)**	0.76 (0.48–1.19)	0.82 (0.52–1.30)	**0.46 (0.30–0.75)**

Adjusted for age, financial readiness, smoking status, alcohol drinkers, and number of medications taken. Values in **bold** are significant.

Group A - none or only 1 of the indicator is low.

Group B - both low HGS & low Physical Performance but not classified as sarcopenic.

## 4 Discussion

This study aimed to determine the prevalence of sarcopenia and its association with quality of life among community-dwelling older Filipinos. Of the 536 participants, it was found that 24.3 % (*n* = 130) had sarcopenia based on the AWGS 2019 criteria, of which 9.3 % (*n* = 50) had severe sarcopenia. Further, the study was able to demonstrate that presence of sarcopenia is associated with a lower self-perceived overall quality of life score of older Filipinos as well as in the physical and psychological health domains, but not in the social relationships and environmental domains. To our knowledge, this is the first in-depth sarcopenia and QOL study in the community setting of the country. This adds valuable scientific knowledge and evidence to the country and Asian regional setting.

### 4.1 Prevalence of sarcopenia

This study used the recent AWGS 2019 criteria in the diagnosis of sarcopenia in both the community and clinical settings [7]. In comparison to previous studies in the Asian region that used similar criteria among community-dwellers (60 years and older), our overall prevalence of 24.3 % is fairly similar to studies conducted among Indonesians, Thai, Chinese, Japanese and Singaporeans that found a prevalence rate of 17.6 %, 22.2 %, 27.1 %, 22.3 % and 32.2 %, respectively [9,[25], [26], [27]–28]. In addition, a study among Koreans aged 70 to 85 years revealed a prevalence of 22.8 % [29]. However, there were also studies that reported higher prevalence of sarcopenia using the same AWGS 2019 criteria. Among Taiwanese older adults (aged 65 and above) admitted to daycare centers, Chang et al. revealed that half of the older persons were confirmed to have sarcopenia [30]. Meanwhile, in a study of Chang et al. among rural community-dwellers in Korea, it was found that 41.0 % had sarcopenia [31].

Locally, our result is higher than prior reports on Filipino older population. The study of Gabat et al. which found a prevalence of 6.1 % among Filipino adults that were outpatients of a tertiary hospital in Manila [32]. The difference in prevalence may be explained with the lower mean age in the study of Gabat in comparison to our study population (60.3 vs 68.1). Furthermore, the discrepancy in the prevalence may also be attributed to the criteria used. Our study used the AWGS 2019 criteria, while Gabat et al. applied the proposed local cut-off criteria for Filipinos from Tee et al. [33] which has lower hand grip strength cut-off than the AWGS 2019 criteria: <24.54 kg and <16.10 kg for male and female, respectively. Likewise, the proposed local criteria for muscle were higher than the AWGS 2019 with <12.5 kg/m^2^ for males and <8.33 kg/m^2^ for females. Hypothetically, If the criteria of Tee et.al. were applied in our study, a lower sarcopenia prevalence would have been observed. Thus, the development and use of local reference value for sarcopenia has been suggested in order to take into account possible differences in ethnicity and environmental factors [19,33]. The criteria of Tee et al. were valued as the first proposed criteria for Filipinos [33]. However, the potential limitations was the sole use of the BIA method in deriving the muscle index criteria and not DXA, a known gold standard for body composition analysis and absence of sensitivity and specificity study employing the proposed criteria against the standard set by the AWGS 2019. It is recommended to revisit the applicability of local and regional criteria to better assess the presence of sarcopenia. Still, our motivation of using the AWGS 2019 criteria was for the purposes of comparability of results among other Asian countries.

HGS is one of the key criteria for sarcopenia. A noteworthy observation found is the high prevalence of low HGS (72.2 %) in our study. Other countries such as Thailand (41.6 %) and Taiwan (51.0 %), also found a high prevalence of low HGS as compared to their overall prevalence of sarcopenia [29,34]. Normative values of hand grip strength reported by Afable et al. from the data of the Longitudinal Study of Ageing and Health in the Philippines (LSAHP) showed a decreasing trend in HGS with increase in age [35] which is in line with the normative data for HGS done in other Asian populations [36]. A plausible explanation for the high prevalence of low HGS is that Filipinos have generally lower statures when compared to other Asian populations [37]. This may have contributed to the observed lower hand grip strength in this study since stature, hand size, is associated with grip strength [38,39]. Kim et al. recommended adjusting HGS with height since the correlation between the two was found to be the strongest [40]. The importance of considering height adjustment in the interpretation of HGS results was also highlighted by Mendes et al. [41]. This underscores the need in coming up with sarcopenia cut-off points accounting for ethnicity and stature as deemed applicable to the country. Moreso, the practicality of hand-grip strength assessment would complement the calf circumference to be a cost-effective initial screening tool in resource-limited setting. On another note, studies

In addition, our findings revealed that the mean age of sarcopenic group (72.3 ± 11.3) is significantly higher than the non-sarcopenic group (66.8 ± 7.6) and that the prevalence of sarcopenia is higher among male (31.2 %, 65 out of 208) than females (19.8 %, 65 out of 328). Advancing age is a primary risk factor for sarcopenia, with studies showing that sarcopenia prevalence increases significantly with age in Asian populations [42]. Further, Male sex has also been observed as an independent risk factor, with men more likely to develop sarcopenia compared to women, attributed to differences in hormonal and muscle mass [43]. In line with other studies, we found that the mean BMI of the sarcopenic group (20.7 ± 3.9) is significantly lower than the non-sarcopenic group(25.2 ± 3.9). BMI is closely linked to sarcopenia, as low body weight can impact decreased muscle mass and strength. However, it should be recognized the potential presence of sarcopenic obesity. A condition where the combination of muscle weakness and overweight and obesity are present [44]. This highlights the complexity between body composition and sarcopenia risk in older adults.

### 4.2 Sarcopenia and QOL

This study demonstrated that QOL in the sarcopenia group was lower, as indicated by the decreased odds in attaining higher physical, psychological, and overall QOL scores as compared to the non-sarcopenia group. This is congruent with other studies who found a similar association between sarcopenia and quality of life [14,[45], [46]–47]. In the study by Sun et al., it was revealed that sarcopenic Korean older adults had significantly lower scores in EuroQOl-5 dimension (EQ-5D) as compared to non-sarcopenic, suggesting a significant association between sarcopenia and HRQOL [13]. Another study conducted by Smith et al. on pooled data of low- and middle-income countries showed a significant association between severe sarcopenia and lower QOL scores which may be explained by factors such as functional impairment and disability related with sarcopenia [48].

Further, our results is congruent to the previous findings that decreasing muscle mass and physical performance were significantly associated with declines in physical QOL among older adults [49,50]. These detrimental age-related changes on muscle mass, strength, and physical function brought about by sarcopenia highlights its profound effect on the daily living and psychological well-being of an older individual and should be taken into account in achieving the goal of healthy aging. However, no significant association was found between sarcopenia and the social and environmental domains. The WHOQOL-BREF intends to assess the perceived personal relationships and social support of an individual for the social domain while the perceived financial resources, freedom, physical safety and security, home environment, opportunities for acquiring new information and skills, recreation or leisure activities, and physical environment for the environmental domain [24,51,52]. In contrast to the physical and psychological domain, the social and environmental domains are highly influenced by the emotional state and outlook in life which may not directly correlate with physical health. Older adults’ accumulated experiences gained over time may have allowed them to develop a sense of confidence and security to overcome a crisis or inconveniences [53]. This is evident in the study of Callueng et al. which found that Filipino adults who are older tend to have greater levels of perceived resilience in times of adversity [54]. Collectively, this may explain the absence of association between sarcopenia and environmental and social domains of the QOL. Nonetheless, ongoing monitoring of these domains remains crucial for a holistic care and well-being of a person.

While we used WHOQOL-BREF to assess the overall QOL of an individual in a broader sense, disease-specific questionnaire such as the Sarcopenia Quality of Life (SARQoL) can be relevant for individuals diagnosed with sarcopenia due to a more focused domains on Physical and mental health, Locomotion, Body Composition, Functionality, Activities of Daily Living, Leisure activities and Fears.

A study in the UK found that SARQoL has acceptable performance and sufficiently responsive for use in clinical studies and monitoring of individual with sarcopenia [55].

It is recommended to come up with a locally-validated version of the SARQoL for use in clinical setting in the country.

### 4.3 Strengths and limitations

Our study has several key strengths. The use of AWGS 2019 criteria for sarcopenia diagnosis ensures diagnostic accuracy, and the use of a culturally-validated WHOQOL-BREF questionnaire enhances the reliability of QOL assessments in this study population. Additionally, all researchers underwent standardized training, ensuring consistency in data collection following a standardized protocol. Also, we conducted an additional stratified analysis, categorizing non-sarcopenic individuals into Group A (reference group) and Group B and still found consistent results, strengthening our conclusions.

However, our study also has some limitations. Self-reported data may introduce selection and recall biases, potentially affecting the accuracy of certain variables. Additionally, the absence of advanced imaging techniques (such as DXA or MRI) limits the diagnostic precision of sarcopenia, as compared to the more practical bioelectrical impedance analysis (BIA). The cross-sectional study design prevents causal inferences. Furthermore, data collection occurred during the COVID-19 pandemic, providing unique insights into the health challenges of older Filipinos at that time but potentially limiting its applicability to normal conditions.

The inclusion of diverse urban geographic sites improves the generalizability of our findings within the urban Philippine context. However, future studies should expand to both urban and rural settings to ensure broader applicability. Additionally, increasing the sample size would strengthen the statistical power and reduce variability in estimates, addressing the relatively wide confidence intervals observed in our results. Despite these limitations, our study provides meaningful findings for public health and aging research in the Philippines as well as to other low and middle income countries. While complete randomization in our study was not feasible due the time period of collection, we increased the sample size to account for potential selection bias and enhance the validity of our conclusions.

### 4.4 Implications of the findings and future direction

Philippines is one of the many countries that is currently considered to have young population but is foreseen to experience a demographic shift in the coming decades. Our findings provide enlightenment on the need to consider the early screening and diagnosis of sarcopenia in community settings as well as in clinical setting. Integrating routine muscle mass and strength assessments into primary healthcare and geriatric care can facilitate early identification of at-risk individuals, potentially preventing severe functional decline, eventually affecting the QOL. The psychological burden of sarcopenia highlights the necessity to ensure accessibility of mental health support alongside prevention or rehabilitation of physical function.

Targeted interventions, including protein-rich nutritional programs and physical activity or resistance training, may mitigate muscle loss and preserve functional independence in older adults alongside psychological interventions to ensure holistic well-being of this population. Policy efforts, in both public health and clinical practice perspective, should integrate sarcopenia prevention into the health and nutrition strategies for the older population in order to promote healthy and independent aging. This will primarily benefit and help prepare countries that will experience demographic shift in the near future.

Future research should explore the longitudinal impact of sarcopenia on quality of life (QOL) and the efficacy of interventions in preventing muscle deterioration in the local setting. In addition, exploring the influence of co-morbidities, physical activity, and diet on sarcopenia progression is recommended. In addition, researches on comparing various sophisticated imaging-based assessments (e.g., DXA scans) against a more practical method (e.g., BIA) could enhance diagnostic accuracy in the local population and encourage cross-country comparability. Further, we recommend to have the SARQoL translated to the local language and be validated for clinical and community use. In resource-limited settings such as the Philippines, prioritizing sarcopenia in research and public health discussions and implementing community-based screening programs are essential. Revisiting local diagnostic criteria and incorporating population-specific adjustments, such as handgrip strength normalization by stature, may improve detection.

## 5 Conclusions

This study highlights a 24.3 % prevalence of sarcopenia among community-dwelling older Filipinos. Presence of sarcopenia is associated with reduced physical health, psychological health and overall QOL scores among Filipino older adults.

## Ethics approval and consent to participate

The project received an ethical clearance from the Food and Nutrition Research Institute Institutional Ethics Review Committee (FIERC No. 2021–017). Signed informed consent was sought from each participant after thorough explanation of the project. The study was conducted in accordance with the Declaration of Helsinki.

## Consent for publication

Not applicable

## Availability of data and materials

The data that support the findings of this study are available from the authors upon reasonable request and with permission of the DOST-FNRI.

## Funding

The data used in the study was from the DOST Grants-in-Aids program funded project titled “Relationship of Body Composition to the Functional Capacity and Quality of Life of Older Filipino in Selected Provinces in the Philippines” with the DOST- Philippine Council for Health Research and Development as the monitoring agency.

## CRediT authorship contribution statement

**Robby Carlo Tan:** Writing – review & editing, Writing – original draft, Validation, Resources, Project administration, Methodology, Investigation, Funding acquisition, Formal analysis, Data curation, Conceptualization. **Kyler Kenn Castilla:** Writing – review & editing, Writing – original draft, Formal analysis, Data curation. **Michael Serafico:** Writing – review & editing, Writing – original draft, Supervision. **Marco Mensink:** Writing – review & editing, Writing – original draft, Supervision, Conceptualization. **Lisette de Groot:** Writing – review & editing, Writing – original draft, Supervision, Conceptualization.

## Declaration of competing interest

All authors declare that they have no known competing financial interests or personal relationships that could have appeared influence the work reported in this paper. RCAT reports financial support for the research was provided by the Republic of Philippines Department of Science and Technology Grants in Aid. The funder had no role in study design, data collection and analysis, decision to publish, or preparation of the manuscript.
